# Values of blockchain for risk-averse high-tech manufacturers under government’s carbon target environmental taxation policies

**DOI:** 10.1007/s10479-022-05030-6

**Published:** 2022-11-04

**Authors:** Tsan-Ming Choi

**Affiliations:** Centre for Supply Chain Research, University of Liverpool Management School, Chatham Building, Liverpool, L69 7ZH UK

**Keywords:** Blockchain, Mean-variance analysis, Data, High-tech products, Risk averse, Carbon target

## Abstract

Today, high-tech industries such as consumer electronics commonly face government rules on carbon emissions. Among the rules, carbon emission tax as well as extended producer responsibility (EPR) tax are two important measures. Using blockchain, the policy makers can better determine the carbon target environmental taxation (CTET) policy with accurate information. In this paper, based on the mean-variance framework, we study the values of blockchain for risk-averse high-tech manufacturers who are under the government’s CTET policy. To be specific, the government first determines the optimal CTET policy. The high-tech manufacturer then reacts and determines its optimal production quantity. We analytically prove that the CTET policy simply relies on the setting of the optimal EPR tax. Then, in the absence of blockchain, we consider the case in which the government does not know the manufacturer’s degree of risk aversion for sure and then derive the expected value of using blockchain for the high-tech manufacturers. We study when it is wise for the high-tech manufacturer and the government to implement blockchain. To check for robustness, we consider in two extended models respectively the situations in which blockchain incurs non-trivial costs as well as having an alternative risk measure. We analytically show that most of the qualitative findings remain valid.

## Introduction

In high-tech manufacturing for products such as consumer electronics, mobile phones, and computers, in addition to research and development (R&D) investments (Wei et al., 2021) and industrial agglomeration (Yang et al., 2022), environmental sustainability is definitely a crucial matter. Many high-tech manufacturers have already paid full attention to reducing emissions.^1^ Forbes.com indicates that firms such as Apple, HP, Fairphone, Microsoft, and Samsung are all sustainable high-tech manufacturers.^2^ For instance, in South Korea, Samsung is a top manufacturer for smart phones and it works with suppliers which are certified by Eco Partner to be environmentally sustainable. In Asia, the high-tech industrial manufacturing giant TSMC is also known to put strong emphasis on green management.^3^ TSMC’s manufacturing has a careful plan for pollution control and waste management.^4^ In the US, Apple is committed to achieve carbon neutral by 2030.^5^ As a matter of fact, extended producer responsibility (EPR) is a well-established policy (Cai & Choi, 2021; Choi & Siqin, 2022) imposed on high-tech industries to govern the end of life products’ future. Mobile phones, personal computers, printers, etc. are examples in which EPR commonly applies in which the manufacturers of these high-tech products (we call them the “high-tech manufacturers” in this paper) need to bear some responsibility regarding the end of life product handling and the corresponding expenses.

At the same time, governments all around the world are considering implementable measures to ensure decarbonization or carbon neutralization. For instance, EU (The European Union) has committed to the carbon neutral scheme which aspires to remove carbon emissions (in the same quantity as it creates) by 2050.^6^ In the US, the government has set the 2030 “Greenhouse Gas Pollution Reduction Target”, which aims to achieve a 50–52% reduction of total emissions (compared to 2005) in 2030.^7^ In order to achieve the carbon targets, governments around the world can consider imposing the well-designed environmental taxes on the manufacturers, especially in the high-tech industries.

In this paper, we consider the case in which the high-tech manufacturer is risk averse (Chen et al., 2007; Choi & Zhang, 2022; Guo & Liu, 2020; Zhang et al., 2022). This assumption on risk aversion is well-supported by the prior literature as high-tech industries face a high level of risk owing to the specific industrial characteristics (see, e.g., Birge, 2000; Van Mieghem, 2003). Being risk averse is indeed a very natural behavior for high-tech manufacturers. In this paper, we examine how this risk aversion attitude will affect the setting of government’s carbon target environmental taxation (CTET) policy under the mean–variance framework (Choi et al., 2018b; Wei & Choi, 2010; Wen & Siqin, 2020).

In addition, today’s business is operating in the digital era (Liu et al., 2022b). Among many disruptive technologies in the digital age (Choi et al., 2022), such as big data analytics (Choi & Lambert, 2017; Choi et al., 2018a) and various other types of information technologies, the use of blockchain (Chowdhury et al., 2022; Shi et al., 2021; Song et al., 2022) is critical. Blockchain (Dutta et al., 2020; Liu et al., 2022a), as a distributed ledger technology, can achieve a few important functions for high tech supply chains. According to Infosys.com, high-tech manufacturers commonly require the availability of good information, which cannot be taken for granted; blockchain can step up to help overcome this challenge by providing high quality and accurate information.^8^ Blockchain is especially useful for consumer electronics manufacturers because of the high complexity associated with the corresponding production processes. Using blockchain can provide synchronized high quality data to support operations.^9^In this paper, we want to focus on blockchain's performance in providing accurate information for the government to set the CTET policy. While if it is absent, we explore the corresponding challenges and uncover when it is beneficial for the high-tech manufacturers to adopt blockchain.

Motivated by the importance of CTET policies and blockchain on high-tech industries, we construct formal models with a goal to analytically study the research questions listed below:

1. What is the form of CTET policy on a risk-averse high-tech manufacturer in order to achieve the carbon target?
2. What is the value of using blockchain for the high-tech manufacturer? When will the use of blockchain be especially beneficial to the manufacturer?
3. How robust are the findings when we consider the costing factors of blockchain and using an alternative risk measure for risk aversion (such as semi-deviation of profit or kurtosis of profit)?

To address the critical research questions raised above, we follow the classic literature (e.g., Erkoc & Wu, 2005; Meijer et al., 2022) and employ the newsvendor problem to help build the theoretical model for the high-tech manufacturer’s production quantity problem. Exploring the above questions yields several insights. For instance, we analytically show that the CTET policy simply relies on the setting of the optimal EPR tax (but not the carbon footprint tax). This finding holds true because of the risk averse attitude of the high-tech manufacturer. Then, in the absence of blockchain, we consider the case in which the government does not know the manufacturer’s degree of risk aversion and then derive the expected value of using blockchain for the high-tech manufacturer. We reveal the conditions under which the use of blockchain is especially useful for the high-tech manufacturer. In our extended models, we investigate the case with blockchain operating costs, as well as the case when the high-tech manufacturer’s risk aversion is changed to be measured by the semi-deviation of profit or kurtosis of profit. We show that most qualitative findings remain the same and hence confirm the robustness of our theoretical findings. The only exception in findings appears for the case when the costs of operating blockchain are positive; in such a case, whether it is wise to implement blockchain does depend on the government’s chance of estimating the high-tech manufacturer’s degree of risk tolerance accurately in the absence of blockchain, which is different from the case when the blockchain operating costs are zero. Managerial implications are discussed (see Sect. 5).

This paper is organized as follows. We concisely introduce the problem and topic in Sect. 1. We conduct a critical literature review in Sect. 2. We build the analytical basic model and study the cases with and without blockchain in Sect. 3. We extend the basic model with considerations of blockchain operating costs as well as different risk measures in Sect. 4. We conclude in Sect. 5 with a discussion on research insights, implications and future research directions. A notation table is present in Appendix (A2) and all technical derivations and proofs are relegated to Appendix (A3).

## Literature review

The following research areas are closely related to this study. We concisely review them as follows.

### Related studies in operations

The high-tech industries are important in the operations literature. Prior studies have examined the procurement problem for parts in the high-tech industry (Zhang et al., 2021), resources management and strategies (Smirnov et al., 2021), logistics systems planning (Lee & Dong, 2008) and assessing the efficiency and performance of the high-tech industry in practice (An et al., 2020). Among other topics, sustainability in high-tech industries deserves our attention and it is also related to this paper. In particular, e-wastes and extended producer responsibility are two hot topics. Plambeck and Wang (2009) analytically explore the impact of e-waste policy on operations. The authors focus their attention on studying the challenge associated with the introduction of new season electronics products. Mazahir et al. (2019) study whether the e-waste rules in Europe are correctly set. In green supply chain operations, Li et al. (2020a) conduct an empirical study on sustainable supply chain operations. The authors uncover the role played by being responsive to changes. Li et al. (2020b) explore green production practices in the apparel industry and examine the related performance measures. More recently, Li et al. (2021) investigate how supply chain contracts can be applied for green products. The authors consider the importance of using marketing means to enhance the demand for green products.

In environmental sustainability, circular supply chain management is proposed (Farooque et al., 2022; Luthra et al., 2022) and controlling emissions (especially carbon emissions) is an important issue, which has also attracted lots of attention over the past decade. For instance, Ma et al. (2018) theoretically investigate the optimal pricing decisions for supply chains in the presence of a carbon emission taxation policy. Chan et al. (2020) explore the use of all kinds of environmental taxations in supply chains with risk-averse retailers. The authors consider in the supply chain context with consumer returns and different contractual arrangements. Sun and Li (2021) discuss the optimization problem for reducing emissions with the proper use of technologies. In this paper, we also consider the presence of carbon emission tax. But different from prior studies, we show that its significance is not as good as the EPR tax when the high-tech manufacturer is risk-averse. As a remark, other related studies include the exploration of a novel concept called extended consumer responsibility (Sheu & Choi, 2019), and the impact of proper merchandise design with a goal to enhancing sustainability (Shi et al., 2016). Note that sustainability also relates to a big area called “closed-loop supply chain management” and reverse logistics (Li et al., 2019). Since this paper does not focus on it, we refer interested readers to Souza (2013) for an excellent review.

Next, we review the use of blockchain (Choi et al., 2019; Hastig & Sodhi, 2020; Yang, 2019) in production and operations. In the supply chain management literature, Choi (2019) analytically investigates the implementation of blockchain for the jewelry supply chain. To be specific, the author explores the value of using blockchain for authentication of diamonds. Choi and Luo (2019) study the use of blockchain to improve quality of information for green supply chain operations. By assuming the normally distributed demand, the authors study when the government should provide sponsors to encourage blockchain deployment in the supply chain. Fan et al. (2020) look into the problem from the consumer perspective on when it is beneficial to implement blockchain in supply chains. Guo et al. (2020) examine the use of blockchain to improve information transparency so that sustainable operations for the apparel supply chain can be achieved. Zheng et al. (2020) explore the smart contracting scheme using blockchain for supply chain operations. Choi (2020a) studies blockchain based initial coin offering for projects with R&D and new product development. Choi (2020b) examines the use of blockchain for financing operations in the newsvendor supply chain. Under the Nash bargaining framework, the author examines the impact brought by blockchain on supply chain contracts. Cai et al. (2021) examine the cheating problems in supply chain contracts and propose the use of blockchain to help.

For service platforms, Choi et al. (2020a) investigate the use of blockchain to support rental platform operations. The authors consider the function of blockchain in allowing truthful information sharing. Choi et al. (2020b) consider the case with an on-demand platform. The authors propose the application of blockchain as a way to estimate the risk attitudes of consumers and evaluate the impacts.

Under COVID-19, Choi and Shi (2022) explore the use of blockchain to enhance “on-demand ride-hailing service platform” operations. The authors uncover the value of blockchain in reducing consumers’ worries of infection.

Recently, the use of blockchain has drawn the attention on a related field called cybersecurity. For example, Rathore and Park (2020) study the “blockchain-based deep learning” approach to improve cybersecurity. Luo and Choi (2022) analytically establish stylized models to study how the use of blockchain based security enhancement scheme can improve cybersecurity and affect government’s penalty scheme. For more research related to the use of blockchain in the operations literature to deal with risk, refer to Choi (2022).

Similar to the above reviewed studies on the use of blockchain in business operations, this paper focuses on revealing how blockchain’s function on obtaining the truthful information of the high-tech manufacturer may affect the environmental taxation policy of the government as well as when it is wise to implement blockchain. From this sense, this idea is similar to the on-demand service platform paper by Choi et al. (2020b). However, the scope and models are totally different between this paper and Choi et al. (2020b).

### Contribution statement

To the best of my knowledge, this is the first paper which highlights the role of blockchain in high-tech manufacturing when the manufacturer is risk averse. As we will see, many novel insights and interesting results are obtained. They have good implications to high-tech manufacturers on their choice of whether or not to proceed with blockchain under the commonly seen government’s environmental taxation policy. Moreover, all results are derived in closed-form and robustness checking has been conducted with extended modelling analyses. Thus, the theoretical findings are all analytically proven and scientifically solid.

## Basic model

### With blockchain

We consider the case in which in a market, the government implements a carbon target environmental taxation (CTET) policy with two parts, namely the per quantity output carbon emission tax *s* and the per unit product sold end of life EPR tax $\xi$. In the CTET policy, there is a carbon target, which can be transferred into a quantity target *Q* for the high-tech manufacturer (see Appendix A1). Note that (i) The quantity target *Q* is different from the conventional carbon emissions target (which is measured in weight units), and (ii) the specific value of *Q* depends on the government’s preference, and the industrial features (e.g., the high-tech manufacturer’s cost revenue parameters and levels of pollution). It is in fact the social welfare maximizing quantity. In Appendix (A1), we demonstrate how to analytically determine the optimal *Q* for the government in our model setting. Note that the optimal *Q* is independent of the high-tech manufacturer’s risk averse preference as the government only focuses on social welfare which includes the environmental impact and profitability of the business operations.

In the following, we consider the case when *Q* is pre-determined (e.g., following the result in Appendix (A1)) for the corresponding high-tech manufacturer. The objective of the government now is to set the CTET policy so as to ensure the high-tech manufacturer can achieve the target *Q*. Note that we do not consider the situation when the government wants to minimize the target *Q* because this is not optimal and will hurt the economic benefit associated with social welfare.

For the high-tech manufacturer, it produces a highly seasonal product and sells it in a short season (i.e., it is a single period problem). The selling price is *r* per unit (i.e., the unit revenue), the production cost is *w* per unit. For product leftover, it incurs a unit holding cost *l* and carries a unit value *v*. Under the government’s CTET policy, each unit of production quantity has to bear an additional carbon emission tax of *s*. Each unit of product sold will be charged with an EPR tax of $\xi$. Demand for the high-tech product *x* is random and follows a density function $f(x)$ and cumulative distribution function $F(x)$. Here, we do not confine the specific form of distribution for $F( \cdot )$ and it can be the uniform distribution, normal distribution, gamma distribution, beta distribution, etc. We define $F^{ - 1} ( \cdot )$ as the inverse function of $F( \cdot )$. The high-tech manufacturer is strictly risk-averse and it needs to make an optimal decision on the production quantity *q*. Obviously, this is a standard newsvendor problem in which the high-tech manufacturer is the “newsvendor”.^10^

However, owing to the risk-averse attitude of the high-tech manufacturer, the optimization objective is not solely on expected profit maximization. To be specific, we employ the mean-variance theory (Chiu & Choi, 2016; Choi et al., 2008) and establish the optimization problem for the high-tech manufacturer in the following:

Problem (MV) Max $\overline{\Pi }(q)$

Subject to $$V[\Pi (q)] \le \lambda^{2}$$,

where $\Pi (q)$ is the high-tech manufacturer’s profit function,$\overline{\Pi }(q)$ is the expected high-tech manufacturer’s profit function, $V[\Pi (q)]$ is the variance of high-tech manufacturer’s profit function, and $\lambda$ is the high-tech manufacturer’s risk tolerance level which reflects its degree of risk aversion.

Problem (MV) is formulated following the risk averse inventory problems’ literature (see, e.g., Wei & Choi, 2010; Chiu & Choi, 2016; Chiu et al., 2018; Choi, 2021) under the mean-variance (MV) framework (to capture risk averse preference of the high-tech manufacturer). Under the MV framework, which is originally from finance, we make use of the variance as an analytical measure for risk. Thus, in Problem (MV), the high-tech manufacturer aims to maximize it profitability (as quantified by the expected profit) and put the level of risk (as quantified by the variance of profit) under “control” by imposing the analytical constraint. Note that in the extended model, we will consider other risk measures and demonstrate the validity of findings under them.

For the sequence of decision making, we consider the situation when the government first determines the production quantity “target” by maximizing social welfare and then sets the CTET policy. After that, the high-tech manufacturer reacts and determines the optimal production quantity which maximizes Problem (MV).

We now present the detailed mathematical expressions. First, following the standard newsvendor problem, we can easily list out the profit function of the high-tech manufacturer as follows:

1 $$\Pi (q) = (r - w - s - \xi )q - (r + l - v - \xi )S(q)$$

where $S(q) = \max (q - x,0)$, which represents the amount of product leftover when the season ends.

Taking expectation of (1), we yield the high-tech manufacturer’s expected profit function in the following:

2 $$\overline{\Pi }(q) = (r - w - s - \xi )q - (r + l - v - \xi )\overline{S}(q)$$

where $$\overline{S}(q) = E[\max (q - x,0)] = \int_{0}^{q} {F(x)dx}$$.

Taking variance of (1), we have the high-tech manufacturer’s variance of profit given in (3):

3 $$V[\Pi (q)] = (r + l - v - \xi )^{2} \Phi (q)$$

where $$\Phi (q) = V[\max (q - x,0)] = 2\int_{0}^{q} {(q - x)F(x)dx} - \left[ {\int_{0}^{q} {F(x)dx} } \right]^{2}$$.

Note that the form of variance of profit in (3) is well-known in the literature even though we have added the EPR tax in (3). From the literature (see Chiu & Choi, 2016), we know the structural properties of (2) and (3). To be specific, $\overline{\Pi }(q)$ is known to be a strictly concave function with a unique peak. The expected profit maximizer is given by the standard critical fractile solution by solving the first order condition $d\overline{\Pi }(q)/dq = 0$: $q_{M}^{*} = \mathop {\arg }\limits_{q} \{ d\overline{\Pi }(q)/dq = 0\} = F^{ - 1} [(r - w - s - \xi )/(r + l - v - \xi )]$. For the variance of profit (3), it is a monotonic increasing function in *q*.

In order to ensure the high-tech manufacturer is risk averse, we consider the case when the constraint $V[\Pi (q)] \le \lambda$ is binding, which is translated into the situation when $V[\Pi (q_{M}^{*} )] > \lambda$ (which means the expected profit maximizing quantity (for the case when the high-tech manufacturer is risk neutral) can never be optimal). The MV efficient region is hence given by $\Omega = (0,q_{M}^{*} )$ in which $q > 0$ (we rule out the trivial case with zero quantity) and $q < q_{M}^{*}$.

In this section, we consider the presence of blockchain so that the high-tech manufacturer’s degree of risk aversion can be accurately estimated (Cai et al., 2020; Choi et al., 2020b) and known by the government. Note that blockchain has many different functions, including smart contracting, cryptocurrency, etc. and can be classified into different categories (e.g., public blockchain, permissioned blockchain, etc.) In this paper, to focus on the core information accuracy issue, we intentionally do not consider these other functions. Of course, one may argue that to have accurate estimate of the high-tech manufacturer’s degree of risk aversion does not necessarily require the use of blockchain, which is true. However, the argument here is that using blockchain can fully support information transparency and it can let the government know the high-tech manufacturer’s degree of risk aversion which is the feature we would like to examine. This is in line with prior studies such as Choi et al. (2020b). In addition, in this section, we intentionally do not include the costs associated with blockchain because (i) the cost of blockchain can be negligibly small when the technology becomes more mature, (ii) the most heavy cost of blockchain is on the technology investment which is a sunk cost, and (iii) we can directly quantify the benefit of having information transparency and then estimate whether it will justify for the investment in the technology. Nevertheless, we have considered the case in which different operating costs for blockchain are present in the extended model and we will see that considering the blockchain cost interestingly does not make much significant difference (except one finding, see Proposition 6) in our core insights and implications.

For a notational purpose, we define: $\phi (q) = \sqrt {\Phi (q)}$, and $\phi^{ - 1} ( \cdot )$ is the inverse function of $\phi ( \cdot )$. Note that since $\Phi (q)$ is monotonic increasing, $\phi (q)$ is also monotonic increasing. $\phi^{ - 1} ( \cdot )$ exists and we assume it has a one to one mapping with its argument. Proposition 1 characterizes the optimal production quantity for Problem (MV).

#### Proposition 1.

*Under a given CTET policy of the government, the risk-averse high-tech manufacturer’s optimal production quantity is given by:*

4 $$q_{MV}^{*} = \phi^{ - 1} [\lambda /(r - v + l - \xi )]$$

Proposition 1 is an important and interesting finding. It not only gives the analytical closed-form expression of the optimal production quantity under Problem (MV), but it also highlights the factors which affect the optimal quantity. To be specific, we can see that the risk-averse high-tech manufacturer’s optimal production quantity will increase if (i) the unit revenue, or the unit holding cost decreases, or (ii) the salvage value increases, or the EPR tax increases, or (iii) the high-tech manufacturer’s degree of risk tolerance increases (i.e., being less risk averse). Note that the above findings are different from the case when the high-tech manufacturer is risk neutral. For instance, when the high-tech manufacturer is risk neutral, its optimal production quantity is given by: $q_{M}^{*} = F^{ - 1} [(r - w - s - \xi )/(r + l - v - \xi )]$, which is larger if the unit revenue increases (rather than decreases under the case with a risk-averse high-tech manufacturer). Moreover, both the per unit production cost *w* and carbon emission tax *s*, which affect the risk neutral high-tech manufacturer’s optimal production quantity plays no role for the case when the high-tech manufacturer is risk averse. We have Proposition 2.

#### Proposition 2.

*For the government, facing the risk averse high-tech manufacturer, the CTET policy to achieve the carbon target is a single EPR taxation policy (i.e., controlling* $\xi$ *is sufficient) and the optimal EPR tax is given as follows:*

5 $$\xi_{MV}^{*} = r + l - v - \lambda /\phi (Q)$$

Proposition 2 is an important finding. First, it highlights the fact that for the case with a risk averse high-tech manufacturer, the CTET policy is simply an EPR taxation policy, and the carbon emission tax *s* is unimportant. Second, it analytically gives the relationship between the optimal EPR taxation rate and the other critical factors, namely the unit revenue, unit holding cost, unit salvage value, degree of risk tolerance and carbon target. In particular, we can see that the optimal EPR tax will be higher if (i) The unit revenue or unit holding is higher, or (ii) the unit salvage value or the degree of risk tolerance is lower, or (iii) the carbon target is higher. Similar to our explanation for Proposition 1, note that the above relationships are different when the high-tech manufacturer is risk neutral. To be specific, if the high-tech manufacturer is risk neutral, then the CTET policy should include both the carbon emission tax *s* and the EPR tax. Moreover, focusing on the case when the government only controls the EPR tax (and keeps *s* as a constant), the optimal EPR tax rate (denoted by $\xi_{M}^{*} |s$; for the risk neutral high-tech manufacturer’s case) is analytically given in closed-form by $\xi_{M}^{*} |s = [(1 - F(Q))r - w - s + (v - l)F(Q)]/[1 - F(Q)]$. Thus, $\xi_{M}^{*} |s$ is higher if the unit salvage value is higher or the unit holding cost is lower, which is just the opposite to the case when the high-tech manufacturer is risk averse. This means that considering the risk averse attitude makes a big difference in terms of shaping the CTET policy.

### Without blockchain

In Sect. 3.1, we consider the situation when the use of blockchain can let the government estimate the level of risk aversion of the high-tech manufacturer accurately (i.e., knowing the real value of $\lambda$). In this section, we consider the case when blockchain is absent and the government only has a probability estimation for $\lambda$. Note that for a given EPR tax rate, the high-tech manufacturer reacts by making the optimal production quantity decision following the optimization problem in Sect. 3.1 (i.e., Problem (MV)).

To be specific, we consider the case in which the government only has a probability $\alpha$ of getting the right estimate for $\lambda$, and a probability $(1 - \alpha )$ of getting an inaccurate estimate for $\lambda$ which we denote as $\hat{\lambda }$ (where $\hat{\lambda } \ne \lambda$). With this information in mind, when will it be wise for the high-tech manufacturer to implement blockchain?

To address this issue, we need to explore the value of blockchain by comparing between the expected profitability under the case with blockchain and the case without blockchain.

From (2) in Sect. 3, in the presence of blockchain, we can easily find that the expected profit of the high-tech manufacturer at the optimal production quantity is given as follows (i.e., we set $q_{MV}^{*} = Q$):

6 $$\overline{\Pi }^{B*} = \overline{\Pi }(q_{MV}^{*} ) = (r - w - s - \xi_{MV}^{*} )Q - (r + l - v - \xi_{MV}^{*} )\overline{S}(Q)$$

where $$\overline{S}(Q) = E[\max (Q - x,0)] = \int_{0}^{Q} {F(x)dx}$$.

In the absence of blockchain, when the government accurately estimates the degree of risk tolerance of the high-tech manufacturer, then the expected profit is equal to $\overline{\Pi }(q_{MV}^{*} )$. Note that this case carries a chance of $\alpha$. When the government wrongly estimates $\lambda$ to be $\hat{\lambda }$ (where $\hat{\lambda } \ne \lambda$), the chance is $(1 - \alpha )$. Combining the two cases, in the absence of blockchain, the expected profit of the high-tech manufacturer at the optimal production quantity is given as follows:

7 $$\overline{\Pi }^{NB*} = \alpha \overline{\Pi }(q_{MV}^{*} ) + (1 - \alpha )[(r - w - s - \hat{\xi })\hat{q} - (r + l - v - \hat{\xi })\overline{S}(\hat{q})]$$

where

8 $$\hat{\xi } = r + l - v - \hat{\lambda }/\phi (Q)$$

9 $$\hat{q} = \phi^{ - 1} [\hat{\lambda }/(r - v + l - \hat{\xi })]$$

From (7), we can clearly see that the expected profit of the high-tech manufacturer depends on the government’s setting of the EPR tax. A wrong estimation of the high-tech manufacturer’s degree of risk tolerance will yield an expected profit of the high-tech manufacturer which may be higher or lower than the correctly estimated one. This is a tricky situation.

### Impacts of using blockchain

With (6) and (7), we can define the expected value of blockchain in (10):

10 $$EVB = \overline{\Pi }^{B*} - \overline{\Pi }^{NB*}$$

After simplification, we can express *EVB* as follows:

11 $$EVB = (1 - \alpha )\{ [(r - w - s - \xi )Q - (r + l - v - \xi )\overline{S}(Q)] - [(r - w - s - \hat{\xi })\hat{q} - (r + l - v - \hat{\xi })\overline{S}(\hat{q})]\}$$

With the derived EVB, suppose that it is wise for the high-tech manufacturer to implement blockchain if and only if $EVB > 0$. Thus, we have Proposition 3a.

#### Proposition 3a

*For the risk-averse high-tech manufacturer, when the costs of operating blockchain are zero: (i) Implementing blockchain is not always a wise decision. (ii) Whether it is wise to implement blockchain is independent of the government’s chance of estimating the high-tech manufacturer’s degree of risk tolerance accurately in the absence of blockchain (i.e.,* $\alpha$*). (iii) Implementing blockchain is more likely to be a good idea if the target Q is larger.*

Proposition 3a gives several important and interesting findings. First, Proposition 3a(i) highlights the fact that for the case with a risk averse high-tech manufacturer, even if there is no additional cost associated with the use of blockchain, it is not always beneficial to use the blockchain. In other words, the high-tech manufacturer should not blindly vote for using blockchain as it may bring more harm than good to itself. Second, from Proposition 3a(ii), we can see that counter-intuitively, the government’s chance of estimating the high-tech manufacturer’s degree of risk tolerance correctly (without using blockchain) is found to be unimportant in deciding whether it is wise or not for the high-tech manufacturer to adopt blockchain. Note that this result does depend on the assumption here that blockchain does not incur additional costs. As we will see in the extended model, this result will not hold if there are blockchain operating costs. Third, from Proposition 3a(iii), we learn that whether it is a good idea to deploy blockchain does relate to the government’s target *Q*. Facing a larger *Q*, the high-tech manufacturer is more likely to vote for using blockchain.

Now, we turn to the government. We employ the social welfare function as established in Appendix (A1) for the following analysis. In the presence of blockchain, the optimal social welfare is given as follows:

$$SW_{{}}^{B*} = SW(Q) = (1 - \eta )EP(Q) - \eta EI(Q)$$

In the absence of blockchain, the social welfare becomes the following:

$$SW_{{}}^{NB*} = \alpha [(1 - \eta )EP(Q) - \eta EI(Q)] + (1 - \alpha )[(1 - \eta )EP(\hat{q}) - \eta EI(\hat{q})]$$

We can define the expected value of blockchain for social welfare as follows:

$$EVB_{SW} = SW^{B*} - SW^{NB*}$$

After simplification, we can express $EVB_{SW}$ as follows:

$$\begin{gathered} EVB_{SW} = (1 - \alpha )\{ (1 - \eta )[(r - w)Q - (r + l - v)\overline{S}(Q)] - \eta [\beta Q + \gamma \overline{S}(Q)] \hfill \\ \;\;\;\;\;\;\;\;\;\;\;\;\;\;\;\;\;\; - [(1 - \eta )[(r - w)\hat{q} - (r + l - v)\overline{S}(\hat{q})] - \eta [\beta \hat{q} + \gamma \overline{S}(\hat{q})]]\}  \hfill \\ \end{gathered}$$

With the derived $EVB_{SW}$, suppose that the government will prefer the use of blockchain if $EVB_{SW} > 0$. We have Proposition 3b.

#### Proposition 3b

*For the government (from the social welfare perspective), when the costs of operating blockchain are zero: (i) Implementing blockchain is not always a wise decision. (ii) Whether it is wise to implement blockchain is independent of the government’s chance of estimating the high-tech manufacturer’s degree of risk tolerance accurately in the absence of blockchain (i.e.,* $\alpha$*). (iii) Implementing blockchain is more likely to be a good idea if the target Q is larger.*

Proposition 3b is very similar to Proposition 3a. The interpretation is similar and hence we skip the full details here.

## Extended models and analyses

In the basic model (Sect. 3), we have made some modelling assumptions. In order to drill deeper and test for robustness of the findings, we consider two extended cases in this section. The details are presented one by one in the following.

### Model C: considering blockchain operating costs

In Sect. 3, we do not consider the operating costs associated with blockchain. In this sub-section, we consider the case when operating blockchain incurs non-trivial costs. We call the model under this case Model C. To be specific, we consider the scenario in which using blockchain requires a per transaction operating cost *c*, as well as a per season fixed cost *J*. With these costs, we reformulate the optimization problem under Model C to be the following.

Problem (C-MV) Max $\overline{\Pi }_{C} (q)$

Subject to $$V[\Pi_{C} (q)] \le \lambda^{2}$$,

where $\Pi_{C} (q)$ is the high-tech manufacturer’s profit function,$\overline{\Pi }_{C} (q)$ is the expected high-tech manufacturer’s profit function, $V[\Pi_{C} (q)]$ is the variance of high-tech manufacturer’s profit function, under Model C. The detailed analytical expressions are given below.

12 $$\Pi_{C} (q) = (r - c - w - s - \xi )q - (r - c + l - v - \xi )S(q) - J$$

13 $$\overline{\Pi }_{C} (q) = (r - c - w - s - \xi )q - (r - c + l - v - \xi )\overline{S}(q) - J$$

14 $$V[\Pi_{C} (q)] = (r - c + l - v - \xi )^{2} \Phi (q)$$

Similar to the basic model’s case, the expected profit maximizing production quantity under Model C can be easily found by solving the corresponding first order condition: $q_{C,M}^{*} = \mathop {\arg }\limits_{q} \{ d\overline{\Pi }_{C} (q)/dq = 0\} = F^{ - 1} [(r - c - w - s - \xi )/(r - c + l - v - \xi )]$. We also focus on exploring the case when the constraint $V[\Pi (q)] \le \lambda$ is binding, which means $V[\Pi_{C} (q_{C,M}^{*} )] > \lambda$.

Similar to Proposition 1, we find that for a given CTET policy of the government, the risk-averse high-tech manufacturer’s optimal production quantity under Model C is given by:

15 $$q_{C,MV}^{*} = \phi^{ - 1} [\lambda /(r - c - v + l - \xi )]$$

For the government, facing the risk averse high-tech manufacturer, the CTET policy to achieve the carbon target is also a single EPR taxation policy and the optimal EPR tax is given below:

16 $$\xi_{C,MV}^{*} = r - c + l - v - \lambda /\phi (Q)$$

Note that the results in (15) and (16) are basically the same as the findings summarized in Propositions 1 and 2, except now the unit operating cost for blockchain is present. However, the main qualitative conclusion remains the same.

Similar to (6), under Model C, we can easily find that the expected profit of the high-tech manufacturer at the optimal production quantity:

17 $$\overline{\Pi }_{C}^{B*} = \overline{\Pi }_{C} (q_{C,MV}^{*} ) = (r - c - w - s - \xi_{C,MV}^{*} )Q - (r - c + l - v - \xi_{C,MV}^{*} )\overline{S}(Q) - J = \overline{\Pi }^{B*} - J$$

#### Proposition 4

*Under the case when the high-tech manufacturer is risk averse, the expected profits under the basic model and Model C at the optimal production quantity and CTET policy are the same if the fixed operating blockchain cost J is zero.*

Proposition 4 is a surprising result because conventional wisdom will indicate that in the presence of a strictly positive per unit operating cost for blockchain, even though the fixed cost of operating blockchain is zero, the expected profit achieved by the risk averse high-tech manufacturer should be smaller than the case when the per unit operating cost for blockchain is zero. The trick behind this result goes to the optimal EPR tax $\xi_{C,MV}^{*}$. As a matter of fact, the presence of *c* reduces the unit revenue while it also directly reduces the optimal EPR tax $\xi_{C,MV}^{*}$ (which aims to entice the high-tech manufacturer to achieve the carbon target). In this situation, the expected profit remains unchanged if the fixed operating cost *J* is zero.

Since Model C considers the presence of blockchain costs, its analysis is devoted to the case when blockchain is present. For the case without blockchain, the results in Sect. 3.2 are valid. Thus, we can now jump to the analysis on the value of using blockchain.

Similar to (11), under Model C, we can derive the expected value of blockchain ($EVB_{C}$) for the high-tech manufacturer to be the following:

18 $$EVB_{C} = EVB - J$$

With (18), we can see that the expected values of blockchain under the basic model and Model C are basically the same except the presence of the fixed blockchain operating cost *J.* As such, we can see that Proposition 3(i) and Proposition 3(iii) are true. However, Proposition 3(ii) is no longer valid because whether it is wise to implement blockchain does depend on the government’s chance of estimating the high-tech manufacturer’s degree of risk tolerance accurately in the absence of blockchain (i.e., $\alpha$). We have Proposition 5.

#### Proposition 5

*For the risk-averse high-tech manufacturer, when the costs of operating blockchain are positive (i.e., under Model C), whether it is wise to implement blockchain does depend on the government’s chance of estimating the high-tech manufacturer’s degree of risk tolerance accurately in the absence of blockchain (i.e.,* $\alpha$*).*

Note that for Proposition 5, the main difference is brought by the presence of the fixed blockchain operating cost *J*. The per unit operating cost plays no role as we can see from Proposition 4’s findings. For the government (and social welfare), similar analyses can be conduced and similar findings as the ones under the case for the manufacturer can be found. We hence do not repeat here.

### Other risk measures

In Sects. 3 and 4, we focus on the case when the risk averse decision of the high-tech manufacturer is formulated under the MV framework. This is in line with the literature (see Chiu & Choi, 2016; Choi et al., 2019). However, variance of profit is not the only measure which can be used to capture risk. In fact, variance of profit has been criticized as a far from perfect measure because variance by definition includes both upside and downside variations which arguably does not really capture the inherent property of risk (which should only cover the downside deviation, i.e., having profit less than the mean). Thus, the downside risk measure such as semi-deviation of profit has been proposed as a risk measure (Chan et al., 2020; Choi & Chiu, 2012). In addition, the high-tech manufacturer may have other risk concern associated with a higher moment such as kurtosis (Zhang et al., 2020). Kurtosis of profit measures the outlier of profit. Having a small kurtosis of profit avoids facing the risk of having extreme values of profit. For the definitions, from Choi and Chiu (2012) and Zhang et al. (2020) we have the following analytical expressions for the two alternative risk measures, and then we present Proposition 6.

The semi-deviation of profit (Choi & Chiu, 2012) for the high-tech manufacturer is:

$$SD[\Pi (q)] = (r + l - v - \xi )^{2} {\rm Z}(q)$$,

where $${\rm Z}(q) = \int_{0}^{{q - \int_{0}^{q} {F(x)dx} }} {(q - \int_{0}^{q} {F(x)dx} - x)f(x)dx}$$.

The kurtosis of profit (Zhang et al., 2020) for the high-tech manufacturer is:

$$K[\Pi (q)] = (r + l - v - \xi )^{4} \Psi (q),$$

where $$\Psi (q)  = 4\int_{0}^{q} {(q - x)^{3} F(x)dx} - 3\left[ {\int_{0}^{q} {F(x)dx} } \right]^{4} + 12\left\{ {\left[ {\int_{0}^{q} {F(x)dx} } \right]^{2} \int_{0}^{q} {(q - x)F(x)dx} - \int_{0}^{q} {F(x)dx} \int_{0}^{q} {(q - x)^{2} F(x)dx} } \right\}$$

#### Proposition 6.

*For the risk-averse high-tech manufacturer, if the risk measure is changed from variance of profit to be semi-deviation of profit or kurtosis of profit, all the qualitative insights in the basic model remain the same.*

Proposition 6 is an interesting result. First, it shows that the findings derived in Sect. 3 are in fact valid not just under the MV framework, but will also hold true if we replace variance of profit by the more perfect measure like semi-deviation of profit (as a downside risk measure) or an alternative measure of kurtosis of profit. This shows the validity of the findings beyond the MV domain.

## Conclusion, insights and future studies

Blockchain is a timely technology. In this paper, we focus on the information aspect of blockchain and consider its presence can allow the government to have correct estimate of the high-tech manufacturer’s risk aversion level. By standard modelling analyses, we have derived many insights and some practical implications. We discuss them in the following one by one.

### Impacts of risk attitude

One of the key focal point of this study is the role played by risk averse attitude. First, we find that the optimal CTET policy depends on the high-tech manufacturer’s risk preference. Being risk-averse is very different from being risk-neutral. To be specific, from Proposition 1 and the discussion thereafter, we note that for the risk-averse high-tech manufacturer, its optimal production quantity will decrease if the unit revenue increases. On the contrary, when the high-tech manufacturer is risk neutral, its optimal production quantity will increase if the unit revenue increases. Moreover, both the per unit production cost and carbon emission tax would affect the risk neutral high-tech manufacturer’s optimal production quantity but they do not affect the risk averse high-tech manufacturer’s optimal production quantity. For the government, facing the risk averse high-tech manufacturer, the CTET policy to achieve the carbon target is a single EPR taxation policy. However, if the high-tech manufacturer is risk neutral, the CTET policy should include both the EPR tax and carbon emission tax. The findings demonstrate that considering risk averse decision making preference is very critical because the results are very different from the case with risk neutral attitude.

### The CTET policy

For the target *Q*, it can be determined by the government by maximizing social welfare (see Appendix (A1)). For the case with the risk averse high-tech manufacturer, the CTET policy is the simple EPR taxation policy. From Proposition 2, we see how the optimal EPR taxation rate relates to other critical factors such as the unit revenue, unit holding cost, unit salvage value, degree of risk tolerance and the carbon target. To be specific, we uncover that the optimal EPR tax will be higher if (i) The unit revenue or unit holding is higher, or (ii) the unit salvage value or the degree of risk tolerance is lower, or (iii) the carbon target is higher. These all give good implications for the government to properly set the EPR policy.

### Implementing blockchain

From Propositions 3a and 3b, we can see that for both the risk-averse high-tech manufacturer and government (with focus on social welfare), when the costs of operating blockchain are zero: (i) Implementing blockchain is not always a wise decision. Thus, even “free lunch” may not be good to both the high-tech manufacturer and the government. (ii) Implementing blockchain is more likely to be a good idea if the target *Q* is larger. Since *Q* = $F^{ - 1} \left( {\frac{(1 - \eta )(r - w) - \eta \beta }{{(1 - \eta )(r + l - v) + \eta \gamma }}} \right)$, a larger *Q* appears when the per unit emission penalty from production (i.e., $\beta$) decreases, or the per unit environmental impact brought by each unit of product sold (i.e., $\gamma$) decreases, or the environmental impact (EI) importance factor decreases. In other words, if the government pays less attention to environmental impact (and hence relatively more on economic benefit), implementing blockchain is more likely to be desirable (when other conditions remain constant). (iii) Whether it is wise to implement blockchain is independent of the government’s chance of estimating the high-tech manufacturer’s degree of risk tolerance accurately in the absence of blockchain. However, for this feature, it does require the condition in which the operating costs of blockchain are zero. If the operating costs of blockchain are non-trivial, then it is no longer true.

### Robustness

(a) Under the case when operating blockchain incurs both a fixed cost and a per transaction cost, when the high-tech manufacturer is risk averse, the expected profits for the high-tech manufacturer under these two cases are the same if the fixed operating blockchain cost is zero (P.S.: Proposition 4). This case happens because the presence of EPR tax will cancel out the operating cost effect in the optimal ordering quantity. Then, from Proposition 5, when the costs of operating blockchain are all positive, whether it is wise to implement blockchain does depend on the government’s chance of estimating the high-tech manufacturer’s degree of risk tolerance accurately in the absence of blockchain. This is an important difference between the major findings under the cases with and without blockchain operating costs. (b) For the risk-averse high-tech manufacturer, if the risk measure is changed from variance of profit to another measure such as the downside risk measure “semi-deviation of profit”, or extreme event measure “kurtosis of profit”, all the qualitative insights derived under the MV model remain the same. This shows the robustness of the findings under the MV model.

In this paper, we focus on the perspective of the risk-averse high-tech manufacturer’s inventory decision and model it using the newsvendor problem. This is supported by the literature. However, we may also extend the problem using other modelling choices. It will also be interesting to extend the analysis to cover the case when remanufacturers are present and examine the interactions between the high-tech manufacturer and the remanufacturer. Contractual arrangements (Chiu et al., 2011) may also be examined. Nowadays, in the sharing economy (Wang et al., 2022), product exchanges are popular. How this affects the results of this study deserves further analyses. Last but not least, supply chain finance is an important research area. Since blockchain has its functions in finance, it will be an interesting extension to include supply chain finance in the high-tech industry and see how that may affect the government’s CTET policies.

## Appendix A1

### Determining *Q*

From the government’s perspective, the consideration of *Q* is to maximize social welfare. Here, we define social welfare as follows,

A1 $$SW(q) = (1 - \eta )EP(q) - \eta EI(q)$$

where

$EP(q)$ is the expected profit generated from the high-tech manufacturer,

$EI(q)$ is the environmental impact (negative) generated from the high-tech manufacturer, and

$0 < \eta < 1$ is the coefficient which represents the relative importance of $EP(q)$ and $EI(q)$ in the mind of the government. We call it the environmental impact (EI) importance factor.

The expression in (A1) is intuitive. The government considers both the economic benefit and environmental impact as well as their relative importance in establishing the analytical social welfare function.

For the detailed analytical expressions, we have the following:

A2 $$EP(q) = (r - w)q - (r + l - v)\overline{S}(q)$$

A3 $$EI(q) = \beta q + \gamma \overline{S}(q)$$

where $\beta > 0$ is the per unit emission penalty from production, and $\gamma > 0$ is the per unit environmental impact brought by each unit of product leftover (with respect to the end of life product handling, etc.)

Thus, putting (A2) and (A3) into (A1), we have:

$$\begin{aligned} SW(q) & = (1 - \eta )[(r - w)q - (r + l - v)\overline{S}(q)] - \eta [\beta q + \gamma \overline{S}(q) \\ & = [(1 - \eta )(r - w) - \eta \beta ]q - [(1 - \eta )(r + l - v) + \eta \gamma )\overline{S}(q) \\ \end{aligned}$$

The government’s objective is to determine the optimal production quantity *q* to maximize $SW(q)$.

Checking the structural properties shows that $SW(q)$ is a concave function of the production quantity *q*. We can hence find the analytical closed-form expression of the optimal production quantity by solving the first order condition:

$$q_{SW}^{*} = \mathop {\arg }\limits_{q} \{ dSW(q)/dq = 0\} = F^{ - 1} \left( {\frac{(1 - \eta )(r - w) - \eta \beta }{{(1 - \eta )(r + l - v) + \eta \gamma }}} \right)$$

Thus, the value of the target *Q* is equal to $F^{ - 1} \left( {\frac{(1 - \eta )(r - w) - \eta \beta }{{(1 - \eta )(r + l - v) + \eta \gamma }}} \right)$.

## Appendix A2

### Notation table

See Table

**Table 1:** Notation

Notation	Details
CTET	Carbon target environmental taxation
EPR	Extended producer responsibility
MV	Mean–variance
*B*	The case with blockchain
*C*	Model C
$\xi$	The EPR tax
*s*	The carbon emission tax
*Q*	Target
$\alpha$	The probability of correctly estimating the high-tech manufacturer’s risk tolerance level
$1 - \alpha$	The probability of incorrectly estimating the high-tech manufacturer’s risk tolerance level
$c$	The per unit operating cost for the case with blockchain under Model C
$r$	The unit revenue
$w$	The unit production cost
*l*	The unit holding cost for end of season leftover
$v$	The unit end of season (salvage) value
*J*	The fixed operating cost of using blockchain under Model C
$\lambda$	The high-tech manufacturer’s risk tolerance level which reflects its degree of risk aversion
$\beta$	The per unit emission penalty from production
$\gamma$	The per unit environmental impact brought by each unit of product leftover (with respect to the end of life product handling, etc.)
$0 < \eta < 1$	The environmental impact (EI) importance factor
$V[.]$	Taking variance
$E[.]$	Taking expectation

1.

## Appendix A3

### All technical derivations and proofs

#### *Proof of Proposition 1*

Under a given CTET policy of the government, the risk-averse high-tech manufacturer aims to determine the production quantity which solve Problem (MV).

Problem (MV) Max $\overline{\Pi }(q)$

Subject to $$V[\Pi (q)] \le \lambda^{2}$$

Note that since $\overline{\Pi }(q)$ is concave and $V[\Pi (q)]$ is monotonic increasing, the optimal production quantity will be bounded between 0 and $q_{M}^{*}$. Since the high-tech manufacturer is considered to be risk-averse and the binding constraint is active $V[\Pi (q)] \le \lambda^{2}$, we have $q < q_{M}^{*}$.

In the solution region, $\overline{\Pi }(q)$ is increasing. As a result, the optimal production quantity is exactly the production quantity which lies on the boundary of the constraint, i.e.,

$$q_{MV}^{*} = \mathop {\arg }\limits_{q} \{ V[\Pi (q)] = \lambda^{2} \}$$

Since $V[\Pi (q)] = (r + l - v - \xi )^{2} \Phi (q)$, we have the following:

$$\begin{gathered} V[\Pi (q)] = \lambda^{2} \hfill \\ \Rightarrow \sqrt {V[\Pi (q)]} = \lambda \hfill \\ \end{gathered}$$

$$\Rightarrow (r + l - v - \xi )\phi (q) = \lambda$$

Rearranging terms yields the optimal production quantity for a given EPR tax:

$$q_{MV}^{*} = \phi^{ - 1} [\lambda /(r - v + l - \xi )]$$ □

#### Proof of Proposition 2

From Proposition 1, for a given $\xi$, we have: $q_{MV}^{*} = \phi^{ - 1} [\lambda /(r - v + l - \xi )]$.

Thus, for the government, facing the risk averse high-tech manufacturer, the CTET policy which can achieve the carbon target is a single EPR taxation policy (i.e., controlling $\xi$ alone is sufficient) and the optimal EPR tax is given as follows.

To achieve the target, we have:

$$q_{MV}^{*} = Q$$

Then, we have:

$$Q = \phi^{ - 1} [\lambda /(r - v + l - \xi )]$$

Rearranging terms yields:

$$\xi_{MV}^{*} = r + l - v - \lambda /\phi (Q)$$ □

#### Proof of Proposition 3a

From the expected value of blockchain for the risk averse high-tech manufacturer in (11), we have:

$$\begin{aligned} EVB & = (1 - \alpha )\{ [(r - w - s - \xi )Q - (r + l - v - \xi )\bar{S}(Q)] \\ & - [(r - w - s - \hat{\xi })\hat{q} - (r + l - v - \hat{\xi })\bar{S}(\hat{q})]\} \\ \end{aligned}$$

Examining $EVB$ closely, we can see that:

(i) $EVB$ need not be positive, and hence implementing blockchain is not always a wise decision.
(ii) Since $0 < \alpha < 1$, we have:
  $$\begin{aligned} EVB & > 0 \Leftrightarrow [(r - w - s - \xi )Q - (r + l - v - \xi )\bar{S}(Q)] \\ &\quad - [(r - w - s - \hat{\xi })\hat{q} - (r + l - v - \hat{\xi })\bar{S}(\hat{q})] > 0 \\ \end{aligned}$$
  Thus, whether it is wise to implement blockchain is independent of the government’s chance of estimating the high-tech manufacturer’s degree of risk tolerance accurately in the absence of blockchain (i.e., $\alpha$).
(iii) $EVB$ is increasing in *Q*. Thus, implementing blockchain is more likely to be a good idea if the target *Q* is larger. □

#### Proof of Proposition 3b

We can check the expected value of blockchain for social welfare:

$$\begin{gathered} EVB_{SW} = (1 - \alpha )\{ (1 - \eta )[(r - w)Q - (r + l - v)\overline{S}(Q)] - \eta [\beta Q + \gamma \overline{S}(Q)] \hfill \\ \;\;\;\;\;\;\;\;\;\;\;\;\;\;\;\;\;\; - [(1 - \eta )[(r - w)\hat{q} - (r + l - v)\overline{S}(\hat{q})] - \eta [\beta \hat{q} + \gamma \overline{S}(\hat{q})]]\}  \hfill \\ \end{gathered}$$

(i) $EVB_{SW}$ need not be positive and hence implementing blockchain is not always a wise decision.
(ii) Since $0 < \alpha < 1$, we have:
  $$\begin{gathered} EVB_{SW} > 0 \hfill \\ \Rightarrow \{ (1 - \eta )[(r - w)Q - (r + l - v)\overline{S}(Q)] - \eta [\beta Q + \gamma \overline{S}(Q)] \hfill \\ \;\;\;\;\;\;\;\;\;\;\;\;\;\;\;\;\;\; - [(1 - \eta )[(r - w)\hat{q} - (r + l - v)\overline{S}(\hat{q})] - \eta [\beta \hat{q} + \gamma \overline{S}(\hat{q})]]\} > 0 \hfill \\ \end{gathered}$$
  Thus. whether it is wise to implement blockchain is independent of the government’s chance of estimating the high-tech manufacturer’s degree of risk tolerance accurately in the absence of blockchain (i.e., $\alpha$).
(iii) Since $EVB_{SW}$ is increasing in *Q*, implementing blockchain is more likely to be a good idea if the target *Q* is larger.□

#### Proof of Proposition 4

From (17), we have:

$$\begin{aligned} \bar{\Pi }_{C}^{{B*}} & = \bar{\Pi }_{C} (q_{{C,MV}}^{*} ) = (r - c - w - s - \xi _{{C,MV}}^{*} )Q - (r - c + l - v - \xi _{{C,MV}}^{*} )\bar{S}(Q) - J \\ & = \bar{\Pi }^{{B*}} - J \\ \end{aligned}$$  .

Directly, observing from it, we can see that under the case when the high-tech manufacturer is risk averse, the expected profits under the basic model and Model C at the optimal production quantity and CTET policy are the same if the fixed operating blockchain cost J is zero.□

#### Proof of Proposition 5

Directly from the expected value of blockchain for the risk averse high-tech manufacturer, we can see that: For the risk-averse high-tech manufacturer, when the costs of operating blockchain are positive (i.e., under Model C), whether it is wise to implement blockchain does depend on $\alpha$. □

#### Proof of Proposition 6

Suppose that we replace the variance of profit by another risk measure $R[\Pi (q)]$. Note that since $\overline{\Pi }(q)$ is concave, if $R[\Pi (q)] \le \lambda$ is active and $R[\Pi (q)]$ is monotonic increasing, the optimal production quantity will be bounded between 0 and $q_{M}^{*}$, and can be found be solving.

$$q_{R}^{*} = \mathop {\arg }\limits_{q} \{ R[\Pi (q)] = \lambda^{2} \}$$

Since $R[\Pi (q)]$ is monotonic increasing, we can find a functional form of the optimal solution similar to $q_{MV}^{*}$.

For the risk-averse high-tech manufacturer, if the risk measure is changed from variance of profit to be semi-deviation of profit or kurtosis of profit, since both semi-deviation of profit (Choi & Chiu, 2012) and kurtosis of profit (Zhang et al., 2020) are monotonic increasing in *q*, the qualitative findings remain the same.□

## Footnotes

1 https://www.accenture.com/us-en/insights/high-tech/sustainability-strategy.

2 https://www.forbes.com/sites/blakemorgan/2020/11/09/10-most-sustainable-consumer-tech-companies/?sh=a4a19bd49a82.

3 https://esg.tsmc.com/en/focus/APractitionerofGreenPower.html.

4 https://esg.tsmc.com/download/file/2018_tsmc_csr_report_published_May_2019/english/pdf/e_6_greenManufacturing.pdf.

5 https://www.apple.com/environment/.

6 https://www.europarl.europa.eu/news/en/headlines/society/20190926STO62270/what-is-carbon-neutrality-and-how-can-it-be-achieved-by-2050.

7 https://www.whitehouse.gov/briefing-room/statements-releases/2021/04/22/fact-sheet-president-biden-sets-2030-greenhouse-gas-pollution-reduction-target-aimed-at-creating-good-paying-union-jobs-and-securing-u-s-leadership-on-clean-energy-technologies/.

8 https://www.infosys.com/about/knowledge-institute/insights/documents/unlocking-blockchain.pdf.

9 https://www.sofocle.com/industry/blockchain-in-electronics/.

10 This paper focuses on EPR and environmental policies which are especially prominent in the “high-tech” industries such as computers and consumer electronics. The use of the newsvendor problem is also traditionally valid to help model the features of these high-tech products, such as volatile demand, and short selling season (see, e.g., Choi et al., 2004; Erkoc and Wu 2005; Meijer et al., 2022).
